# Coronavirus disease-2019 epidemic response in Uganda: The need to strengthen and engage primary healthcare

**DOI:** 10.4102/phcfm.v12i1.2443

**Published:** 2020-06-04

**Authors:** Innocent K. Besigye, Michael Mulowooza, Jane Namatovu

**Affiliations:** 1 Department of Family Medicine, School of Medicine, College of Health Sciences, Makerere University, Kampala, Uganda; 2 Department of Community Health, Jinja Regional Referral Hospital, Jinja, Uganda

**Keywords:** epidemics, primary care, family physician, primary healthcare, epidemic response

## Abstract

In Uganda, the numbers of new coronavirus disease cases have continued to increase slowly since the first case was confirmed. Given that the disease is likely to be holoendemic, the role of primary care (PC) with its features of comprehensiveness, accessibility, coordination and continuity, functioning at the heart of a primary healthcare (PHC) approach, will be important. The elements of PC are applicable in the epidemic preparation, case finding and management, follow-up and post-epidemic phases of responding to this pandemic. This also presents opportunities and lessons for strengthening PHC as well as for reflections on missed opportunities. The effective use of available resources in response to the epidemic should mainly focus on community mobilisation and PHC teams for the prevention, screening, testing and treatment of mild and moderate cases.

## Introduction

The first coronavirus patient was confirmed on 21 March 2020. Before that a great deal of anxiety had been experienced in the country, given that all the neighbouring countries already had confirmed cases. Although expected, this pronouncement by the Ministry of Health worsened the anxiety and created anger amongst the citizens, given that the case was a recent traveller who should have heeded the government warning not to travel.^1^

Since the confirmation of the first case, the number of new cases has continued to rise albeit slowly with a total of 53 cases at the time of writing this article. The threat of coronavirus disease-2019 (COVID-19) is likely to end when a critical number of people develop herd immunity either from a vaccine or from direct exposure. This will flatten the epidemiological curve. The threat is both to the lives and livelihoods of people. This calls into play elements and principles of primary healthcare (PHC) in order to improve and sustain the health of individuals, families and communities. It is likely that the disease will become holoendemic. Therefore, primary care (PC), functioning at the heart of PHC, with its features of comprehensiveness, accessibility, coordination and continuity, will be important.

In Uganda, PC is based on a decentralised district health system. This district-based PC system consists of different levels of health facilities, with each level providing a different range of services. The health system is linked with the community through the village health teams (VHTs) and community health workers (CHWs). This structure and organisation is intended to strengthen PC. More than 80% of the population live within a 5 km radius from the PC health facility. This article explores the need to strengthen and engage PC and PHC in the fight against COVID-19 in Uganda.

### Role of primary care

The importance of PC in the epidemic preparation, response and post-epidemic is clear, given the expected spread of the disease. Strengthening PC services and delivery systems is of paramount importance. However, the approach to preparation for and response to the epidemic has not had PHC at the forefront. It was noticeable within the medical fraternity that senior medical doctors desired a more comprehensive approach that included PHC and not just a focus on hospital-level intensive care:

‘There are not enough ICU [*intensive care unit*] beds already and many (people) now do not have access to ambulatory care that could prevent hospital admission and ICU care.’ (Remark from one senior specialist doctor on the doctors’ WhatsApp forum)

‘My personal view is that more effort should be made on other things not ICU. We need ICU in a system that is functional, COVID or no COVID.’ (A professor’s comment on WhatsApp)

#### Preparation phase

There were ongoing preparations in anticipation of the first cases. Mass sensitisation of the population was attempted at all levels: village or community, district, regional and national levels.

National level health education targeted the prevention of the disease through proper handwashing and personal hygiene. This was spearheaded by the President through regular addresses to the nation.^2,3^ The President emphasised behavioural and cultural change. He encouraged and directed citizens to avoid risky cultural practices like frequent handshaking and mass attendance of funerals and weddings.

The Presidential directives were disseminated through a decentralised system at the district and community levels. District and community leaders were responsible for ensuring that no gatherings happened, public places had water and soap for handwashing and that handwashing happened.

The preparation phase in the health system, however, had a significant focus on high-level intensive care and hospitals. Critical care health staff were trained by the Ministry of Health (MOH) in collaboration with the World Health Organization (WHO). This phase greatly neglected PC providers, who see most patients, particularly those in private practice, who can assist with identifying, testing and referring suspected patients.

Only one laboratory, the Uganda Virus Research Institute (UVRI), is available in Uganda where samples are analysed. Sample collection centres were established in tertiary and district hospitals. No community-based sample collection centres were planned. Therefore, people must travel to hospitals for sample collection and samples must then be transported to the sole laboratory centre. Therefore, laboratory testing is very limited, with a long turnaround time of up to 36 h.

#### Management of cases

All patients who tested positive have been isolated and admitted to hospitals according to WHO recommendations.^4^ All the cases had mild to moderate disease. Home care is not yet being done because patients are still few, and also because of the prevailing panic and anxiety. It was thought that families would not be comfortable having the patients in their homes. Patients were also likely to suffer serious stigma and violence. Before the first case was confirmed, there were instances of community members calling police to investigate their neighbours who were suspected of having the disease.^5^ There was also no certainty that strict self-isolation in the home, to avoid infecting the family and community members, would be feasible.

The whole country went into complete lockdown to minimise the person-to-person contact in order to prevent or minimise community transmission that would overwhelm the available health facilities. However, no PHC re-organisation was done to comprehensively deal with the pandemic and effectively maintain the essential health services.

### Opportunities for primary healthcare strengthening as a result the pandemic

The activities listed below have been done in response to the crisis created by the pandemic. If sustained, they would strengthen PHC because they are clear examples of its principles:

- The pandemic has acted as a catalyst to innovations like the development of a low-cost ventilator by Makerere University researchers for use in hospitals.^6^
- Government provided food to poor people and powdered milk to pregnant women during the lockdown.^7^ Recognition of vulnerable populations and addressing their welfare and health needs are an act of promoting equity.
- Community action for health was demonstrated through donations by non-governmental organisations, religious groups and the business community.^8^
- Political re-awakening more than ever before particularly on health financing is needed. This can be an opportunity to generate and sustain momentum for Universal Health Coverage.^9^
- Inter-sectoral collaboration in the formation of the COVID-19 taskforce that drew members from laboratory, clinical, public health, security and immigration sectors.

### Primary healthcare pitfalls during the pandemic

- Community participation, a principle of PHC, was low, as most of the measures were dictated and enforced by security and military personnel. This scared away the potential patients who would have come for testing and created resentment of the prevention measures.
- Poor inter-sectoral collaboration between the relevant ministries, for example, the Ministry of Works and Transport could not avail permits for movement early enough during the lockdown. As a result, maintaining essential health services became challenging as health workers could not easily move.
- Failure to use the existing systems in communities and PC facilities to identify suspected patients for testing.
- Neglect of the role of CHWs and VHTs in health education. These would have been instrumental in household-level health education, for example, regarding social distancing within the household.

## Recommendations

The pandemic response should focus more on PHC to address the limitations of screening and testing, innovations on isolation at home, management of mild and moderate cases in the community, as well as active case and contact follow-up. This will protect the scarce hospital-based resources for managing severe and critical cases and promote a people-centred response to the epidemic. Effective use of resources during the response is key to sustain an effective health system post-pandemic. Private health providers can play a key role in the response to the pandemic and should not be neglected.

## Conclusion

The response to the epidemic mainly centred around hospitals and higher levels of the health system and focused on treatment of severe cases rather than mobilisation of communities and PHC teams for the prevention, screening, testing and treatment of mild and moderate cases, which were the most common. Preparedness for the epidemic should be part of PHC systems and simulated trainings should be regularly conducted to maintain relevant competences.
